# Biophysical Features of Bacillithiol, the Glutathione Surrogate of *Bacillus subtilis* and other Firmicutes

**DOI:** 10.1002/cbic.201300404

**Published:** 2013-10-02

**Authors:** Sunil V Sharma, Miriam Arbach, Alexandra A Roberts, Colin J Macdonald, Murree Groom, Chris J Hamilton

**Affiliations:** [a] School of Pharmacy, University of East AngliaNorwich Research Park, Norwich NR4 7TJ (UK); [b] ECOspray Limited Grange Farm HilboroughThetford, Norfolk IP26 5BT (UK); [c] School of Chemistry, University of East Anglia, Norwich Research ParkNorwich NR4 7TJ, (UK)

**Keywords:** ionization potentials, NMR spectroscopy, redox chemistry, thiol–disulfide exchange, thiols

## Abstract

Bacillithiol (BSH) is the major low-molecular-weight (LMW) thiol in many low-G+C Gram-positive bacteria (Firmicutes). Evidence now emerging suggests that BSH functions as an important LMW thiol in redox regulation and xenobiotic detoxification, analogous to what is already known for glutathione and mycothiol in other microorganisms. The biophysical properties and cellular concentrations of such LMW thiols are important determinants of their biochemical efficiency both as biochemical nucleophiles and as redox buffers. Here, BSH has been characterised and compared with other LMW thiols in terms of its thiol p*K*_a_, redox potential and thiol–disulfide exchange reactivity. Both the thiol p*K*_a_ and the standard thiol redox potential of BSH are shown to be significantly lower than those of glutathione whereas the reactivities of the two compounds in thiol–disulfide reactions are comparable. The cellular concentration of BSH in *Bacillus subtilis* varied over different growth phases and reached up to 5 mm, which is significantly greater than previously observed from single measurements taken during mid-exponential growth. These results demonstrate that the biophysical characteristics of BSH are distinctively different from those of GSH and that its cellular concentrations can reach levels much higher than previously reported.

## Introduction

**Scheme 1:**
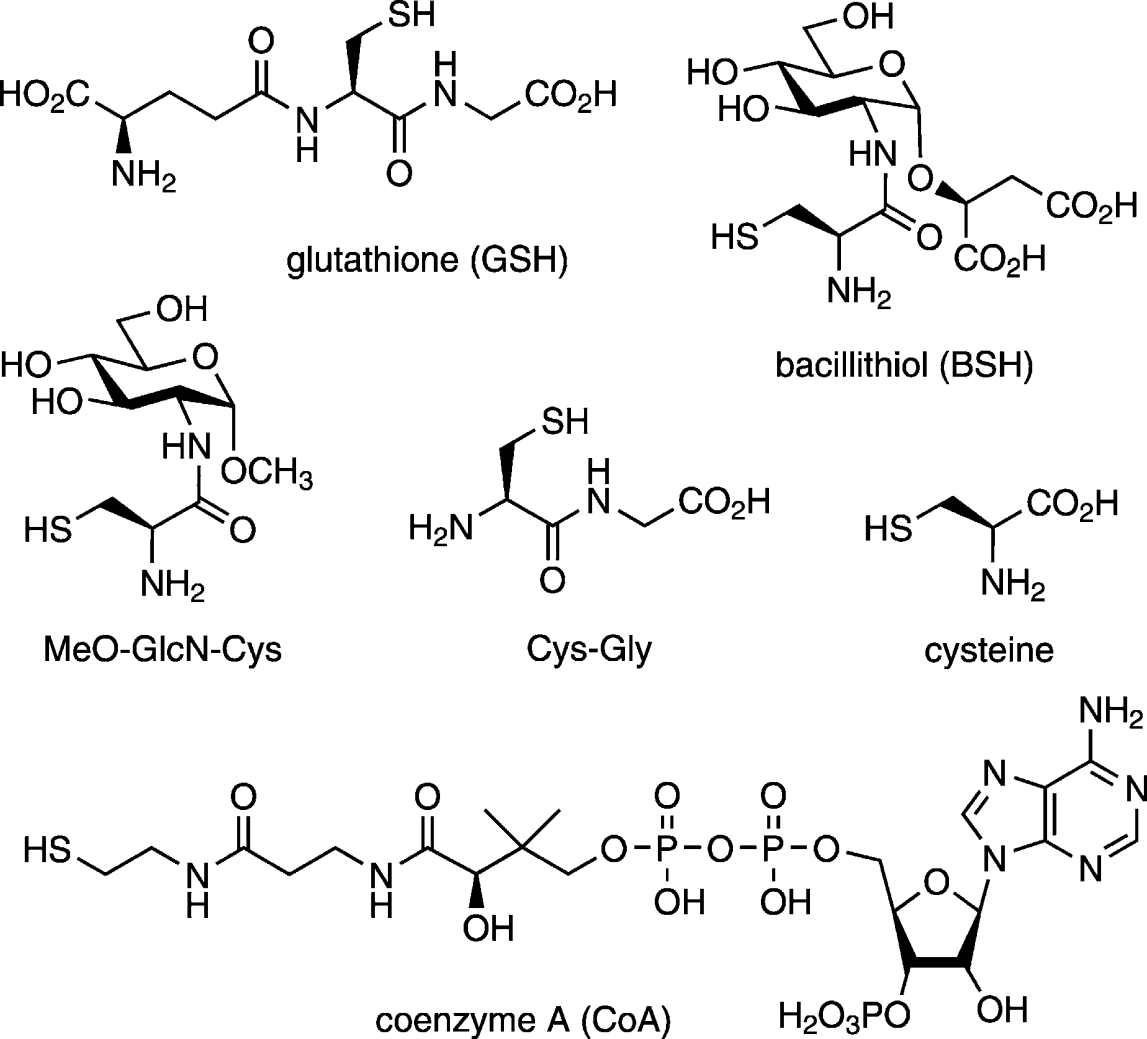
Structures of the LMW thiols relevant to this study.

In eukaryotes and most Gram-negative bacteria, glutathione (GSH, Scheme 1) is the major low-molecular-weight (LMW) thiol cofactor, serving a number of important metabolic functions.^1^, ^2^ GSH plays a critical role in oxidative stress management and in maintaining an intracellular reducing environment. Protein glutathionylation (reversible formation of GS–S-protein disulfides) is also an important post-translational modification for regulating protein function and protecting exposed cysteine (Cys) residues from irreversible damage during oxidative stress.^3^, ^4^ Glutathione-S-transferases mediate the metabolism/detoxification of various electrophilic metabolites/xenobiotics through S-conjugation with GSH, whereas GSH-dependent glyoxalases mediate detoxification of methylglyoxal to lactic acid.^5^

Instead of GSH, many Gram-positive bacteria produce other, structurally distinct LMW thiols, which serve similar metabolic functions to GSH.^6^–^9^ In 2009, bacillithiol (BSH, Scheme 1) was discovered as the predominant LMW thiol in many low-G+C Gram-positive Firmicutes, which do not produce GSH or mycothiol (MSH).^10^ These include bacilli (e.g., *Bacillus subtilis*, *B. anthracis*, *B. cereus*, *B. megaterium*, *B. pumilis*) and some staphylococci (e.g., *Staphylococcus aureus*, *S. saprophyticus*) and streptococci (*Streptococcus agalactiae*). The functions of this recently discovered biothiol, such as its role in detoxification of fosfomycin,^11^ reactive oxidants and methylglyoxal,^12^ as well as the protection and redox regulation of protein function (i.e., protein-S-bacillithiolation during oxidative stress),^13^, ^14^ are now beginning to emerge. As with other LMW thiols,^15^ the thiol p*K*_a_, redox potential and intracellular concentrations of BSH are implicated in its functional efficiency both as a redox buffer and a chemical scavenger of reactive oxidants and electrophiles. Here the fundamental biophysical properties of BSH have been determined and compared with those of other LMW thiols.

## Results

### Thiol, amine and carboxylate p*K*_a_ values

**Figure 1:**
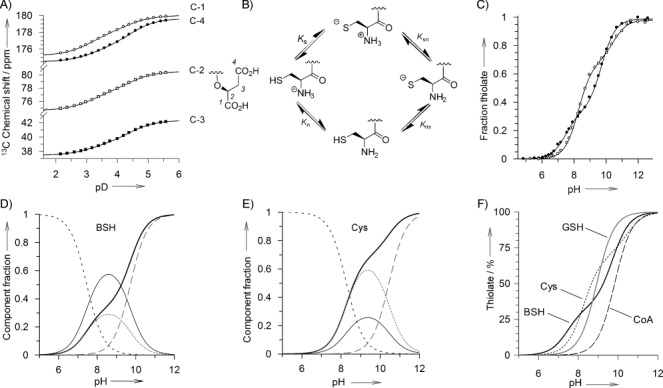
Macro/microscopic p*K*_a_ analyses of BSH and Cys. A) Chemical shifts of the malate carbon atoms of BSH C-1 (○), C-4 (•), C-2 (□), C-3 (▪) as a function of pD. B) The deprotonation pathways that account for the four microscopic acid dissociation constants for a cysteinyl thiol bearing a free amino group. C) pH-dependent thiolate titration curves for BSH (•) and Cys (○). D) and E) The calculated pH-dependent proportions of different thiol/amino protonation forms [HS–NH_3_^+^ (- - - -), ^−^S–NH_3_^+^ (⋅⋅⋅⋅⋅⋅⋅⋅), HS–NH_2_ (—), ^−^S–NH_2_ (– – –) and total thiolate (**—**) of BSH and Cys, respectively. F) The calculated pH-dependent proportions of total thiolate forms of BSH (**—**), GSH (**—**), Cys (⋅⋅⋅⋅⋅⋅⋅⋅) and CoA (– – –).

The macroscopic p*K*_a_ values for the malate carboxylate groups of BSH (p*K*_a1_ and p*K*_a2_) were determined from titration curves showing changes in the ^13^C NMR chemical shifts of the malate carbon atoms in the pD 2.1–5.6 range (Figure 1 A). This provided p*K*_a_ values of 3.14 and 4.38 (Table 1), which are both lower than the corresponding p*K*_a_ values for malic acid (3.40 and 5.13).^16^

**Table 1:** Macroscopic and microscopic p*K*_a_ values of different LMW thiols^[a]^

	Cys	BSH	MeO-GlcN-Cys	Cys-Gly^18^	Cys^18^	GSH^41^	CoA^42^	Malic acid
p*K*_a3_	8.28^[b]^	7.46	7.02		8.33^[b]^			
p*K*_a4_	10.45^[c]^	9.72	9.39		10.7^[c]^			
p*K*_s_	8.38	7.97	7.79	7.87	8.53	8.93	9.83	
p*K*_n_	8.77	7.63	7.10	7.14	8.86			
p*K*_ns_	9.94	9.55	9.31	9.48	10.03			
p*K*_sn_	10.40	9.21	8.62	8.75	10.36			
p*K*_a1_		3.14						3.40^16^
p*K*_a2_		4.38						5.13^16^

[a] For clarity, standard errors have been omitted from this table; however, standard deviations for calculated p*K* values were all <0.09 p*K* units. The data errors are provided in the Supporting Information. [b] Cys only contains a single carboxylic acid group, so this value is p*K*_a2_. [c] Cys only contains a single carboxylic acid group, so this value is p*K*_a3_.

Macroscopic p*K*_a_ values for the thiol and amino groups of BSH and Cys (p*K*_a3_ and p*K*_a4_, Table 1) were determined by measuring the pH-dependent changes in absorbance at 232 nm for the thiolate anion (Figure 1 C).^17^, ^18^ The methyl glycoside derivative of BSH—MeO-GlcN-Cys (Scheme 1)—was also analysed to gain insight into the influence of the BSH malate aglycone on its thiol and amine acid dissociation constants relative to Cys. The macroscopic p*K*_a_ values do not represent the individual (microscopic) p*K*_a_ values of the thiol and ammonium groups, because the p*K*_a_ of each is influenced by the protonation status of the other.^19^ The sequential deprotonation of the thiol and ammonium groups can proceed by two different routes (Figure 1 B). Four different forms of these compounds can therefore exist; their microscopic dissociation constants (p*K*_s_, p*K*_n_, p*K*_sn_ and p*K*_ns_, Table 1) were then calculated from the macroscopic p*K*_a_ and pH-dependent thiolate concentrations as described in Equations (2)–(5) in the Experimental Section.

The values obtained for Cys were comparable to those previously determined by similar procedures.^18^ The Cys microscopic p*K*_a_ values show that the ammonium group is a weaker acid than the thiol by ≈0.4 p*K*_a_ units (i.e., p*K*_n_>p*K*_s_ and p*K*_sn_>p*K*_ns_). The microscopic p*K*_a_ values for BSH were all consistently lower than those determined for Cys. However, contrary to what is observed for Cys, in BSH the ammonium group is more acidic than the thiol by ≈0.3 p*K*_a_ units (i.e., p*K*_n_<p*K*_s_ and p*K*_sn_<p*K*_ns_). Replacing the malate portion of BSH with an uncharged methyl aglycone (MeO-GlcN-Cys) lowers all of the microscopic p*K*_a_ values even further, but the effect is most notable for the microscopic acid dissociation constants for the amino group (p*K*_n_ and p*K*_sn_), which are ≈0.5 p*K*_a_ units lower than those of BSH. This effect could be due to one (or both) of the negatively charged BSH carboxylate groups helping to stabilise the positively charged ammonium group in its protonated form. Consequently, the loss of this stabilising effect in MeO-GlcN-Cys makes the ammonium group more acidic.

The BSH p*K*_a_ values are more comparable to those of Cys-Gly than to those of Cys. Although the inductive effect of the cysteine carboxylate group helps to increase the acidity of the thiol and ammonium groups, this is partially tempered by the electrostatic effect of its negative charge. With both BSH and Cys-Gly the cysteinyl carboxylate is capped with an uncharged amide group, so the inductive effect remains, but the electrostatic effect is removed; hence the acidities of both the amino and thiol functional groups are increased. These observations indicate that, unlike in the case of Cys, the amino and thiol p*K*_a_ values of BSH are more strongly influenced by the amide-linked glucosamine motif than by the malate aglycone. Whereas the acidity of the BSH is comparable to that of Cys-Gly, the compound is significantly more acidic than GSH, which lacks a positively charged amino group on its cysteine residue to help stabilise the thiolate anion.

The microscopic p*K*_a_ values were used to calculate the proportions of the different protonated forms of BSH and Cys over the pH 6–12 range (Figure 1 D and E). The percentage ratios at pH 7.7 are presented in Table 2. A comparison of the pH-dependent proportions of total thiolate forms of BSH, GSH, Cys and CoA is also given in Figure 1 F.

**Table 2:** Distributions of the different protonated forms of BSH, Cys and CoA at pH 7.7

Protonation status	Proportion at pH 7.7 [%]		
	BSH	Cys	CoA
HS–NH_3_^+^	36.5	79.1	–
^−^S–NH_3_^+^	20.9	14.4	–
HS–NH_2_	41.6	6.3	–
^−^S–NH_2_	1.0	0.2	–
total RS^−^	21.9	14.6	0.7

### Thiol–disulfide exchange reactivity

**Scheme 2:**
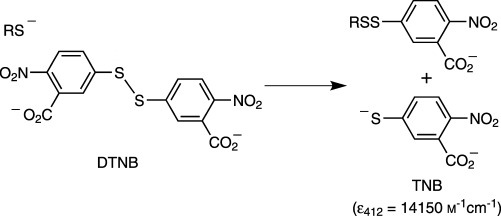
The thiolate reaction with Ellman's reagent used to measure thiol–disulfide exchange reactivity.

Mechanistically, a thiol–disulfide exchange reaction proceeds through a simple S_N_2 displacement reaction between a thiolate anion and a disulfide.^19^ The reactivity of the thiolate nucleophile in such a reaction depends on its acidity and the pH of the reaction buffer. The more acidic the thiol, the higher its thiolate concentration will be at physiological pH. To gauge the relative thiol–disulfide exchange reactivities of the thiolate anions of BSH, Cys and CoA, their pH-independent rate constants (*k*_1_) were determined for reaction with 5,5′-dithiobis-(2-nitrobenzoic acid) (DTNB) as a model disulfide (Scheme 2).

The rate constant determined for GSH (2.02×10^5^ m^−1^ s^−1^, Table 3) was comparable to that previously determined under similar conditions (2.0×10^5^ m^−1^ s^−1^).^20^ Whereas *k*_1_ for CoA was comparable to that of GSH, BSH and Cys were two and four times less reactive, respectively. The pH-independent rate constant of MeO-GlcN-Cys is comparable to that of BSH; this indicates that the carboxylate groups of the malate aglycone do not influence the reactivity of BSH in this thiol–disulfide exchange reaction. Although BSH is more sterically hindered, its pH-independent rate constant is 40 % faster than that of Cys. The relative reactivities of these thiols with other disulfide substrates might differ depending on complementary/repulsive thiol–disulfide binding interactions that could enhance or deplete reactivity accordingly.

**Table 3:** Thiol–disulfide exchange reactivities of LMW thiols with DTNB

Thiol	*k*_1_	[RSH]	[RS^−^]	Physiological rate	
	[s^−1^ m^−1^]	[mm]^[b]^	[μm]^[b]^	[s^−1^×10^6^]^[b]^	relative^[c]^
BSH	(0.95±0.04)×10^5^	1.22	267	2.54	100
CyS	(0.49±0.01)×10^5^	0.20	29	0.14	5.5
CoA	(1.98±0.08)×10^5^	0.46	3	0.06	2.4
GSH^[a]^	(2.02±0.14)×10^5^	10	556	11.2	441
MeO-GlcN-Cys	(0.86±0.03)×10^5^				

[a] A typical intracellular GSH concentration of 10 mm in Gram-negative bacteria has been used here for the purpose of comparison. [b] Based on intracellular pH 7.7 in *B. subtilis*^22^ and cellular thiol concentrations measured during mid-exponential growth (OD_600_=1.40). [c] Relative to the BSH rate normalised to 100.

However, it is worth noting that the fourfold difference in the relative reactivities of Cys and CoA thiolates with DTNB is comparable to the previously reported ninefold difference in their relative reactivities with GSSG.^21^ This suggests that reactivity with DTNB is a reasonable model for comparing the relative thiol–disulfide exchange reactivities of these LMW thiols.

Although the pH-independent thiol–disulfide exchange reactivity of CoA appears to be greater than for BSH, CoA has a much higher thiol p*K*_a_ and lower intracellular abundance than BSH. To illustrate the impact this is likely to have on the relative chemical reactivities of these biothiols in vivo, their *k*_1_ values were corrected for their respective thiolate concentrations during mid-exponential growth and at physiological pH (pH 7.7 for *B. subtilis*;^22^ Table 3). The results of this analysis suggest that, under these physiological conditions, BSH would be 18 and 40 times more chemically reactive than Cys and CoA, respectively. Such differences could vary even more as the relative cellular concentrations of these biothiols change during different stages of growth.

### Bacillithiol redox potential

**Figure 2:**
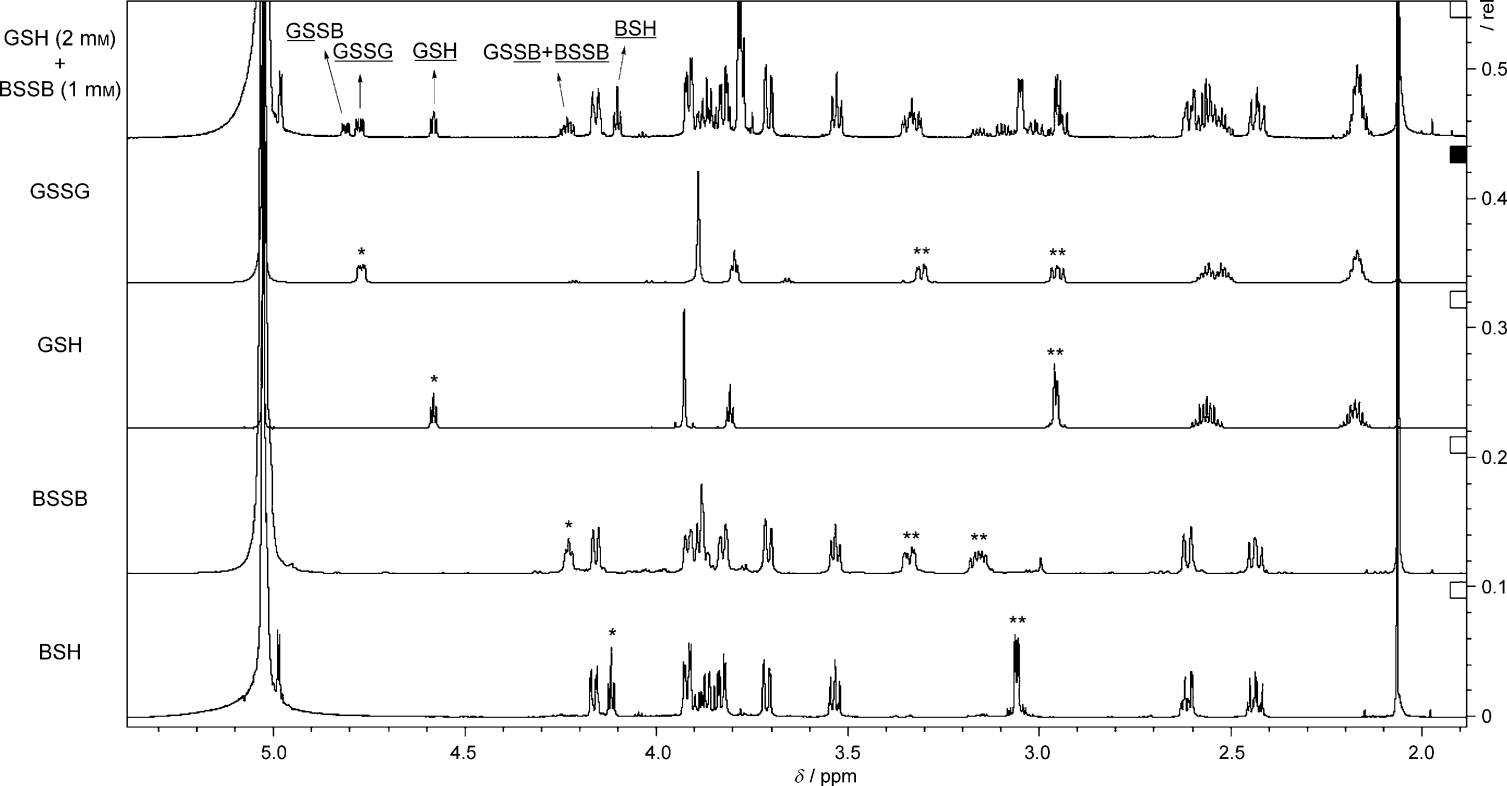
Redox potential determination. Overlay of ^1^H NMR spectra (pD 7.0, 5 °C, 800 MHz) for reaction between BSSB (1 mm) and GSH (2 mm) after equilibrium, together with pure samples of BSH, BSSB, GSH and GSSG. Cysteinyl protons are indicated as * CHα and ** CH_2_β.

The thiol–disulfide redox potential of BSH (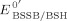) was determined by measuring the thiol/disulfide equilibrium constants for BSH and GSH in both the forward (BSH/GSSG) and reverse (BSSB/GSH) directions. High-field (800 MHz) proton NMR provided sufficient resolution of the resonances associated with the cysteinyl α-protons for BSH, BSSG, GSH and GSSG for their equilibrium ratios to be quantified (Figure 2). At 25 °C, the peaks associated with the α-protons of the cysteine motifs in GSSG and BSSG were obscured by the HOD solvent peak of the equilibration buffer. However, the temperature dependence of the HOD chemical shift^23^ enabled these to be revealed when NMR spectra were measured at 5 °C. Signals for the BSH cysteinyl α-protons in BSSB and BSSG presented a set of overlapping multiplets, so BSSB was quantified indirectly by subtracting the BSSG integral value (4.81 ppm) from the overlapping multiplets (at 4.23–4.29 ppm). These were then used to calculate 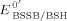 relative to the previously calculated GSH redox potential (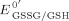
=−240 mV)^24^ by use of the Nernst equation. Thiol redox potentials for Cys and CoA have previously been determined by similar methods,^21^ relative to 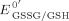
=−205 mV).^25^ Here these literature values have been corrected so they can be compared with 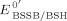, relative to the more accurate 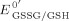 value of −240 mV.^24^ The measured standard thiol redox potential of BSH (−221 mV) is comparable with that of Cys (−223 mV) and higher than those reported for GSH (−240 mV), CoA and γ-glutamylcysteine (−234 mV; Table 4).

**Table 4:** Thiol–disulfide redox potentials (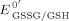)

Thiol	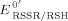	Ref.
GSH	−240	24, 43
γGC	−234	44
CoA	−234^[a]^	21
Cys	−223^[a]^	21
BSH	−221±3	this study

[a] Previously reported values have been corrected relative to 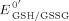
=−240 mV.^24^

### BSH levels in *B. subtilis*

**Figure 3:**
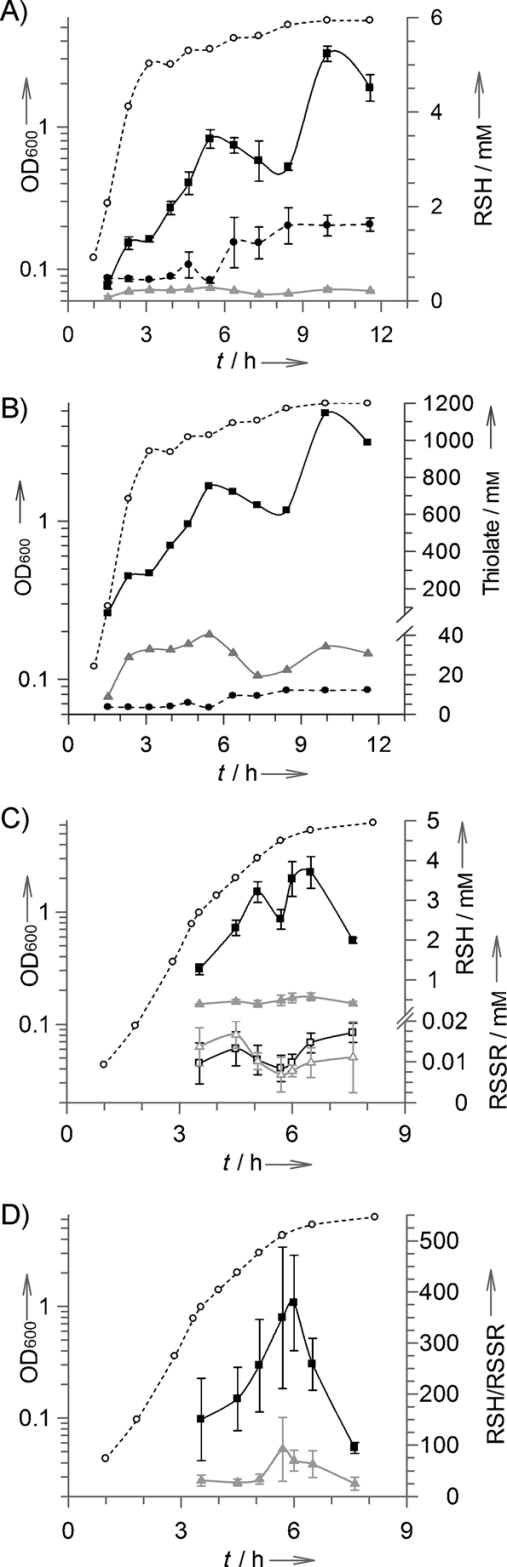
A) Variations in intracellular BSH (–▪–), Cys (–▴–) and CoA (– –•– –) levels* during different growth phases (– –○– –) of *B. subtilis*. B) Thiolate levels* of BSH (–▪–), Cys (–▴–) and CoA (– –•– –). C) Thiol and disulfide levels* of BSH (–▪–), Cys (–▴–), BSSB (□) and (Cys)_2_ (▵). D) Thiol–disulfide redox ratios of BSH/BSSB (–▪–) and Cys/(Cys)_2_ (–▴–). * Intracellular thiol concentrations are derived from the thiol content (μmol g^−1^ rdw) based on previous calculations of 1 μL of intracellular water content per mg rdw in *B. subtilis*^28^ (i.e., 1 μmol g^−1^ rdw=1 mm).

The intracellular LMW thiol and disulfide concentrations were quantified during different stages of growth of *B. subtilis*, from early exponential to late stationary phase (Figure 3 A). LMW thiols were analysed by treating cells with the fluorescent thiol labelling reagent monobromobimane, and the bimane-labelled thiols in the cell extracts were then quantified by HPLC. For disulfide analyses, thiols were first capped by treatment of cells with *N-*ethylmaleimide before reduction of the disulfides (with dithiothreitol), so they could then be quantified after bimane labelling. In *B. subtilis* cultured in LB medium, the intracellular Cys concentration is maintained at a consistently low level (≈0.13–0.28 mm) throughout all stages of growth. The CoA concentration remains relatively constant (≈0.5 mm) during exponential growth, but then increases threefold during stationary phase. Interestingly, the BSH level increases during exponential growth and then temporarily decreases in early stationary phase (OD_600_=3.5–5.2), before rapidly recovering in late stationary phase to a peak value (5.2 mm) ≈17 times higher than for Cys and ≈3.5 times higher than for CoA at the same time point. However, conversion of these thiol levels into thiolate levels at physiological pH shows how cellular concentrations of Cys thiolate exceed those of CoA thiolate by two to tenfold during different stages of growth (Figure 3 B). BSH thiolate levels are consistently higher than the thiolate levels of Cys and CoA by one to two orders of magnitude.

In a separate set of experiments, the thiol/disulfide ratios of BSH and Cys were also determined (Figure 3 C). BSSB and cystine concentrations are maintained between 7–17 μm, and are at their lowest levels as the cells approach stationary phase, when a reduction in BSH is also observed. Throughout the growth curve, the thiol/disulfide ratio for BSH (ranging from 100:1 to 400:1) is consistently higher than that for Cys (25:1 to 90:1) (Figure 3 D). For both thiols, these redox ratios peak during early stationary phase growth.

## Discussion

Unlike in the case of GSH, which in some Gram-negative bacteria can be present in significant excess over Cys (e.g., >200-fold in *E. coli*),^26^ previous thiol analyses of BSH-utilising bacteria have reported much lower levels of BSH that are often equivalent to—or no more than six times higher than—those of Cys.^10^, ^12^, ^13^, ^27^ Most of these reported measurements were only taken from cell culture samples at a single time point, usually during mid-exponential growth. The original analyses of BSH content in *B. subtilis*, as well as a number of other Firmicutes, reported BSH levels of 0.2–0.7 μmol g^−1^ residual dry weight (rdw). The intracellular concentrations of BSH were then estimated on the basis of the global assumption that all of the bacteria analysed contained ≈3 μL of intracellular water per mg of dry weight.^10^ For 0.6 μmol g^−1^ rdw, this equates to an intracellular BSH concentration of approximately 0.2 mm. However, the rdw to intracellular water ratios are not the same in all bacteria. For *B. subtilis* ≈50 % of cell mass has been calculated as being intracellular water,^28^ so a BSH measurement of 0.6 μmol g^−1^ rdw in *B. subtilis* equates to an intracellular concentration of 0.6 mm (i.e., three times higher than previously reported).

Here, *B. subtilis* BSH concentrations have also been shown to increase significantly during exponential growth (Figure 3 A). Collectively, this indicates that BSH levels higher than those previously reported and comparable to GSH levels observed in some Gram-negative bacteria can be attained.^26^ As *B. subtilis* approaches stationary phase there is a temporary decrease in BSH (by ≈1 mm) that cannot be accounted for in terms of a compensatory increase in BSSB. Some of this BSH might be diverted into protein bacillithiolation^13^ to help regulate function during the transition from exponential to stationary phase. Proof of this, and of whether or not such BSH oscillations are observed in other BSH-producing bacteria, remains to be seen. The observed variations in BSH levels in *B. subtilis* do not necessarily represent what will be observed in all other BSH-producing bacteria; recent studies in *S. aureus*, for example, have shown that BSH levels appear to remain constant during exponential growth.^11^ As observed here for *B. subtilis*, a similar decrease in BSH levels is observed during early stationary phase, but those analyses were not continued into late stationary phase, so it is currently not clear if this decrease in BSH levels in *S. aureus* recovers during late stationary phase growth.^11^

The standard redox potential of BSH (−221 mV) is higher than that of GSH (−240 mV), which implies it has a lower capacity to buffer oxidative stress than GSH does. Redox potentials are a thermodynamic property based on thiol–disulfide exchange equilibria. In living cells, however, although the redox status can be maintained in a steady state, it is never at equilibrium.^29^ Coupled with those of Cys and CoA, knowledge of the BSH/BSSB standard redox potential could prove to be a useful parameter for quantifying disturbances in redox metabolism of BSH-utilising microorganisms. However, the actual redox buffering properties of BSH are likely to be driven by other factors (i.e., cellular abundances and catalytic efficiencies of BSH-specific redox enzymes). A number of BSSB reductases have so far been proposed,^10^, ^12^, ^30^ but none of these has yet been isolated and characterised in detail. In *B. subtilis* the BSH/BSSB ratios are always maintained at a high level in favour of BSH (>100:1), and these ratios are always greater than those of Cys/Cys_2_ (Figure 3 C and D).

Interestingly, amongst BSH-producing bacteria, phylogenetic profiling has identified a glutaredoxin-like protein (YphP) as a candidate bacilliredoxin.^12^ YphP also has a remarkably high redox potential (−118 mV)^31^ relative to typical glutaredoxins (−200 mV).^24^ It will be interesting to see whether or not the redox potential differences between BSH and GSH systems are counterbalanced by differences in the kinetics of the enzymes that regulate the intracellular BSH redox status.

During exponential growth, the BSH/BSSB redox ratio continues to increase, but the increases in BSH cannot be accounted for by compensatory reductions in BSSB. The increases in BSH are most likely to arise from BSH biosynthesis, whereas the decreases (in early and late stationary phase) could potentially be accounted for by increased protein bacillithiolation and/or metabolic degradation of BSH. Proof of this, however, remains to be seen.

The thiol group in BSH is more acidic than those in Cys, CoA and GSH (Table 1). At physiological pH (i.e., pH 7.7 in *B. subtilis*),^22^ the percentages of thiolate for BSH, Cys and CoA are 22, 15 and 1 %, respectively (Table 2). The significance of this becomes evident when the cellular LMW thiol concentrations in *B. subtilis* are converted into the corresponding thiolate concentrations at physiological pH (Figure 3 B). This shows that bacillithiolate levels are significantly higher than the thiolate anion concentrations of Cys and CoA. The predominance of the BS thiolate most probably accounts for enhanced chemical reactivity of BSH with different electrophilic biomolecules within the cell.

During oxidative stress, protein thiols can be oxidised to their sulfenic acids. Under these conditions, LMW thiols can react with the sulfenic acids to trap them as mixed disulfides (i.e., protein-SOH+RSH→protein-SSR+H_2_O), preventing further oxidation to the sulfinic and irreversibly damaged sulfonic acid derivatives. Analogously to the role of GSH in Gram-negative bacteria, protein S-bacillithiolation is now emerging as an important thiol redox mechanism for the regulation of protein function during oxidative stress.^13^, ^14^, ^32^, ^33^ In *B. subtilis* the redox-sensitive peroxiredoxin transcription regulator (OhrR) is bacillithiolated during cumene hydroperoxide stress; this suggests a function for BSH in redox sensing.^32^, ^33^ Interestingly, smaller quantities of Cys-OhrR and CoA-OhrR protein mixed disulfides are also observed during mass spectrometric analysis of OhrR purified from cumene-hydroperoxide-stressed *B. subtilis*. To date, 37 different proteins that are S-bacillithiolated in *B. subtilis* and other BSH-producing microorganisms under hypochlorite (NaOCl) stress have been identified.^13^, ^14^ These include several proteins involved in amino acid, cofactor and nucleotide biosynthesis, as well as some translation factors, chaperones and redox proteins. With some of these proteins (e.g., methionine synthase (MetE)) S-cysteinylation has also been observed as a less abundant redox modification that becomes more prominent in BSH-deficient mutants.^13^, ^14^ Although the chemical rates of reaction between LMW biothiols and physiologically relevant disulfides (e.g., cystine, GSSG) are too slow to be of metabolic significance,^21^, ^34^ their chemical reactivities are much faster with more reactive protein sulfenic acids and sulfenyl chlorides (e.g., produced under peroxide and hypochlorite stress), as well as with S-nitrosylated thiols and thiyl radicals, which react to form protein mixed disulfides.^35^ If the physiologically corrected differences in the reactivities of BSH, Cys and CoA with DTNB (Table 3) are mirrored in their reactivities with sulfenic acids and sulfenyl chlorides, this could explain why mixtures of BS-, Cys- and CoA-protein mixed disulfides have been observed in oxidatively stressed *B. subtilis*, with bacillithiolation being the most abundant protein-S-thiolation process in response to oxidative stress.^13^, ^14^ It will be interesting to see whether or not this is observed in other BSH-producing bacteria such as *S. aureus*, in which thiolate concentrations of BSH and Cys are more comparable. Whereas many LMW-thiol-mediated processes are enzyme-catalysed, the detoxification of reactive carbonyl electrophiles (e.g., methylglyoxal) is dependent on an initial nonenzymatic reaction with the LMW thiol to form a hemithioacetal, which, in GSH-utilising organisms, is converted into lactate by the glyoxalase enzymes (Glx-I-II).^36^ Although no BSH-dependent glyoxalases have yet been characterised, BSH null mutants display increased sensitivity to methylglyoxal.^12^, ^30^ Significantly greater intracellular bacillithiolate concentrations suggest that BSH, rather than Cys, would be the preferential reactant with such electrophiles in vivo.

It has been suggested that BSH could function as a metal ion chelator in vivo, due to the proximity of the thiol, amino and malate carboxylate groups.^10^ BSH null mutants display enhanced sensitivity to Cu^2+^, Cd^2+^ and dichromate (Cr_2_O_7_^2−^) metal ion toxicity.^30^ In vitro, BSH is also able to prevent Zn^2+^ activation of the metallothiol-S-transferase FosB by sequestering the divalent metal cation.^11^ At physiological pH, both malate carboxylate groups of BSH are fully deprotonated (i.e., ideal for bidentate metal coordination). At this pH, most of the thiolate in both BSH and Cys resides is associated with a protonated amine (^−^S–NH_3_^+^; Table 2). Of the total BSH, 37 % has both thiol and amino groups protonated (HS–NH_3_^+^; 79 % for Cys), whereas 42 % has an uncharged amine group and a protonated thiol (HS–NH_2_; only 6 % for Cys). The propensity to form these different protonation forms of BSH at physiological pH might influence how effectively it can coordinate metal ions in a mono-, di-, tri- or tetradentate manner. The malate aglycone clearly influences the acidities of the thiol and amino groups of BSH. Therefore, if BSH does prove to play a role in metal ion chelation/trafficking in vivo, it will be interesting to see whether or not metal ions influence the thiol acidity (and potential cellular thiolate concentrations).

## Conclusions

There are clearly some differences between the biophysical characteristics of BSH and those of other, structurally distinct, and more extensively studied, LMW thiols. It will be interesting to see whether and how these are reflected in the unexplored metabolic functions of BSH (e.g., in redox regulation, xenobiotic detoxification, metal ion homeostasis), which are likely to be driven by its chemical and biophysical properties, as well as those of the enzymes that utilise BSH as a substrate or cofactor to mediate such biochemical processes.

## Experimental Section

**General**: BSH^37^ and MeO-GlcN-Cys^11^ were chemically synthesised as described previously. Stock solutions of all thiols were quantified by titration against DTNB (2 mm) in phosphate buffer (pH 7.5) and measurement of the absorbance increase at 412 nm (*ε*=14 150 m^−1^ cm^−1^) due to formation of 5-thio-2-nitrobenzoic acid (TNB).^38^ Accurate stock solutions of DTNB were prepared by quantification of the TNB thiolate anion formed when DTNB was reduced by a large (tenfold) excess of tris-carboxyethyl phosphine (TCEP). Disulfides (BSSB, GSSG) were prepared from titrated quantities of BSH and GSH, which were then oxidised by treatment with aqueous NH_4_HCO_3_ (33 mm) under aerobic conditions as previously described.^37^ All malate carboxylate, thiol p*K*_a_ and thiol–disulfide reaction kinetics data were analysed with the appropriate equations and use of GraFit Version 5 (Erithacus Software, Ltd). NMR spectra were recorded with Varian 800 MHz or Bruker 400 MHz spectrometers. Chemical shifts for NMR are measured in parts per million (*δ*) relative to HOD (5.03 ppm at 5 °C)^23^ with use of internal standards acetonitrile (2.06 ppm) and dioxane (67.19 ppm) for ^1^H and ^13^C NMR, respectively. Coupling constants (*J*) are quoted in hertz (Hz). NMR spectra were recorded at 5 °C for redox titrations and 25 °C for p*K*_a_ determinations. UV absorbance measurements were performed with a PerkinElmer UV Lambda 25 spectrophotometer and quartz cuvettes (1 cm pathlength). The *B. subtilis* CU1065 wild-type strain was generously provided by Prof. John Helmann (Cornell University).

**Carboxylate p*K***_**a**_ **measurements for BSH**: Macroscopic p*K*_a_ values for the malate carboxylate groups of BSH were determined by nonlinear regression analysis of plots of ^13^C NMR chemical shifts of the malate carbons (*δ*_obs_) versus pD with use of Equation (1a), where p*K*_a_ and p*K*_a_′ are the macroscopic acid dissociation constants, and *Lim* is the inflection point on the biphasic curve.


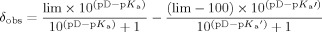
(1a)

pH determinations were performed with an InLab Flex-Micro pH probe and Jenway 3510 pH meter. pH measurements in D_2_O solutions were corrected for the deuterium isotope effect by use of the equation pD=pH+0.40.^39^ A solution of BSH (50 mm in D_2_O) was acidified to pD 2.1 with DCl (1 m). Stepwise increases in pD were achieved by the addition of NaOD (1 m or 200 mm in D_2_O). NMR analyses were carried out at increasing pD values ranging from pD 2.1 to pD 5.6 (at ≈0.2 pD increments). The final macroscopic p*K*_a_ values were the mean values of those determined from titration curves for each of the four carbons on the malate aglycone.

**Thiol and amine p*K***_**a**_ **measurements for BSH, MeO-GlcN-Cys and Cys**: Macroscopic and microscopic p*K*_a_ values were measured by adopting procedures previously described for Cys anion.^17^, ^18^, ^40^ A total of 27 solutions of sodium phosphate buffers (100 mm), ranging from pH 4.85 to pH 12.6 in ≈0.25 pH increments, were prepared. These were made from mixtures of mono-/disodium phosphate (pH 4.85–9.74) and disodium phosphate+NaOH (pH 10.04–12.6). Thiol stock solutions were freshly prepared in ultrapure water on the day of use. All experiments were conducted at 25 °C in quartz cuvettes in a final volume of 1 mL. After blanking of the buffer, the thiol (to a final concentration of 40 μm) was added and rapidly mixed, and the UV absorbance at 232 nm (Abs_232_) was immediately measured to detect the thiolate anion. In control experiments (at pH 12), continuous monitoring of the UV absorbance revealed that the Abs_232_ value decreased by <5 % during the first 10 min under aerobic conditions. This demonstrates that the measurement made immediately after mixing was sufficiently accurate without thiolate measurements being underestimated due to base-catalysed thiol oxidation. The Abs_232_ values at pH 4.85 and 12.6 were normalised to 0 % and 100 % thiolate content, respectively. The macroscopic p*K*_a_ values of the thiol and amino groups of BSH, MeO-GlcN-Cys and Cys were determined by nonlinear regression analysis of a plot of fraction of thiol in its thiolate form (*α*_s_) versus pH fitted to Equation (1b), where p*K*_a_ and p*K*_a_′ are the macroscopic acid dissociation constants, and *Lim* is the inflection point on the biphasic curve.


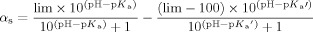
(1b)

For the thiol and amino groups, the microscopic acid dissociation constants (*k*_s_, *k*_n_, *k*_sn_ and *k*_ns_, Figure 1 B) were calculated from Equations (2)–(5):


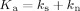
(2)


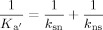
(3)


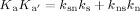
(4)


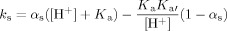
(5)

After Equation (5) had been used to calculate *k*_s_, the other three microscopic constants were calculated from Equations (2) and (4).

**Thiol–disulfide exchange rate constants**: All reactions were carried out at 30 °C in disposable cuvettes (1 mL final assay volume) in sodium phosphate buffer (100 mm) and at pH 4.58 for all thiols that were analysed, except for CoA, for which reactions were conducted at pH 5.54. Reactions were carried out at pH values well below the thiol p*K*_a_ values to ensure that the reactions could proceed at measurable rates. The reactivity of each thiol was monitored at five different concentrations (2–100 μm), and each experiment was carried out in duplicate. Reactions were initiated by the addition of the thiol to a buffered solution of DTNB (40 μm), which was rapidly mixed, and the initial linear rate of TNB production was monitored for 20 s by measuring the increase in absorbance at 412 nm. Under these conditions, the initial rates were measured within the first 2 % of total thiol consumption, allowing the assumption that [RS^−^]_t_=[RS^−^]_0_ and enabling the rate Equation (6) for this reaction:


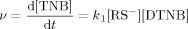
(6)

alternatively expressed as Equation (7):


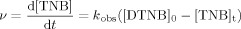
(7)

and subsequently integrated to give Equation (8), where *k*_obs_ is the observed reaction rate (s^−1^) for the specified reaction conditions.


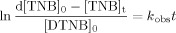
(8)

The *k*_obs_ values at different thiol concentrations were obtained from the linear fit of ln {([DTNB]_0_−[TNB]_t_)/[DTNB]_0_} versus time (*t*). Because *k*_obs_=*k*[RS^−^], a replot of *k*_obs_ versus [RS^−^] was then used to obtain the pH-independent rate constant (*k*_1_).

**Redox potentials**: Deuterated phosphate buffer (50 mm, pD 7.0) was prepared by dissolving monobasic sodium phosphate (0.215 g) and dibasic sodium phosphate (0.133 g) in D_2_O (40 mL). The pH was adjusted to 6.6 by addition of a small amount of solid dibasic sodium phosphate. Acetonitrile (100 μL) was added as an internal standard. The buffer was deoxygenated by purging with dry nitrogen gas. The final volume was adjusted to 50 mL with deoxygenated D_2_O under nitrogen in a glovebox. The resultant buffer was then stored in the glovebox and used for all the thiol–disulfide exchange experiments.

NMR tubes and solid samples of all thiols and disulfides were kept under nitrogen in a glovebox for at least 24 h prior to the preparation of stock solutions in deoxygenated deuterated buffer. Equilibrium mixtures were prepared with appropriate volumes of the corresponding thiol (BSH or GSH) and disulfide (GSSG or BSSB). The volume was then adjusted to 500 μL with deuterated buffer to obtain final thiol/disulfide ratios of 2:1 or 4:1 (mm). These solutions were transferred to separate NMR tubes and allowed to equilibrate in the glovebox for at least 36 h. The thiol/disulfide ratios were monitored by proton NMR every 8–12 h until equilibrium was reached.

Equilibrated thiol and disulfide ratios were determined by integration of their cysteinyl CHα protons, the chemical shifts of which were as follows: BSH (*δ*=4.13 ppm, t, *J*=5.9 Hz), BSSB (*δ*=4.27 ppm, t, *J*=6.4 Hz), GSH (*δ*=4.58 ppm, t, *J*=5.8 Hz), GSSG (*δ*=4.77 ppm, dd, *J*=9.8, 4.3 Hz), GSSB (*δ*=4.81 ppm, dd, *J*=9.9, 4.3 Hz), GSSB (*δ*=4.23–4.29 ppm, m, overlapping the BSSB CHα signal). These values were used to calculate the equilibrium constant [*K*_c_, Equation (9)], which was then used to calculate the BSH redox potential relative to the previously calculated GSH/GSSG redox potential (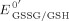
=−240 mV)^24^ by using the Nernst equation [Equation (10)]. In Equation (10), *R* is the gas constant (8.314 J K^−1^ mol^−1^), *F* is the Faraday constant (9.65×10^4^ C mol^−1^), *T* is the absolute temperature (K), and *n* is the number of electrons transferred=2.


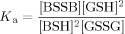
(9)


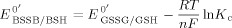
(10)

**Thiol quantification in**­ ***B. subtilis***: *B. subtilis* CU1065 was grown in triplicate cultures in LB medium. The OD_600_ value was monitored, and samples, corresponding to approximately 5 mg or 30 mg (for thiol or disulfide analysis, respectively) rdw of cells, were harvested from each culture at various times. Cell pellets were frozen at −20 °C until derivatisation with monobromobimane (mBBr).

Thiol^13^ and disulfide^26^ analyses were performed as described previously, with some minor modifications (see the Supporting Information). BSmB and CySmB were separated by HPLC as previously described^13^ (Method A; see the Supporting Information). CoAmB was analysed by modification of a previously described method^10^ with use of a shortened gradient (Method B; see the Supporting Information). Detection was carried out with a Jasco fluorescence detector (FP-2020 Plus) with excitation at 385 nm and emission at 460 nm, and a gain of 1×. BSmB and CySmB eluted at 11.8 min and 14.3 min, respectively (Method A). CoAmB eluted at 15.4 min (Method B). All samples were quantified by comparison with BSmB, CysmB and CoAmB standards of known concentration, and the results were converted to μmol RSH g^−1^ rdw. For *B. subtilis*, a thiol quantity of 1 μmol g^−1^ rdw equates to a cellular concentration of 1 mm.^28^

## Supplementary material

As a service to our authors and readers, this journal provides supporting information supplied by the authors. Such materials are peer reviewed and may be re-organized for online delivery, but are not copy-edited or typeset. Technical support issues arising from supporting information (other than missing files) should be addressed to the authors.
