# Supplementary material for: Biophysical Features of Bacillithiol, the Glutathione Surrogate of Bacillus subtilis and other Firmicutes
Source: Chembiochem. 2013 Oct 2;14(16):2160–8. doi: 10.1002/cbic.201300404 (PMC4065351; doi:10.1002/cbic.201300404)
Supplement: Supplementary file 1 [file cbic0014-2160-SD1.pdf]

CHEMBIOCHEM

Supporting Information

© Copyright Wiley-VCH Verlag GmbH & Co. KGaA, 69451 Weinheim, 2013

Biophysical Features of Bacillithiol, the Glutathione Surrogate of *Bacillus subtilis* and other Firmicutes

Sunil V. Sharma,[a] Miriam Arbach,[a, b] Alexandra A. Roberts,[a] Colin J. Macdonald,[c] Murree Groom,[b] and Chris J. Hamilton*[a]

cbic_201300404_sm_miscellaneous_information.pdf

Supporting Information

Contents

Macroscopic and microscopic pK_a values of different LMW thiols	1
^{13}C NMR analysis for malate pK_a determination of BSH. Overlay of selected regions of ^{13}C NMR spectra (100 MHz) at various pD.	2
Plots of rate of TNB product formation (K_{obs}) vs thiolate anion concentration for different LMW thiols with DTNB.	3
Thiol-disulfide equilibrium constants and redox potentials	4
Thiol and disulfide quantification in *Bacillus subtilis*	5

Table S1. Macroscopic and microscopic pK_a values of different LMW thiols

	**Cys**	**BSH**	**MeO-GlcN-Cys**
**pK_{a3}**	8.28 ± 0.02[a]	7.46 ± 0.03	7.02 ± 0.06
**pK_{a4}**	10.45 ± 0.05[b]	9.72 ± 0.01	9.39 ± 0.01
**pK_s**	8.38 ± 0.09	7.97 ± 0.06	7.79 ± 0.06
**pK_n**	8.77 ± 0.08	7.63 ± 0.05	7.10 ± 0.07
**pK_{ns}**	9.94 ± 0.08	9.55 ± 0.06	9.31 ± 0.09
**pK_{sn}**	10.40 ± 0.09	9.21 ± 0.07	8.62 ± 0.08
		**BSH**	
**pK_{a1}**		3.14 ± 0.09	
**pK_{a2}**		4.38 ± 0.05	

[a] Cys only contains a single carboxyl group so this value is pK_{a2}
[b] Cys only contains a single carboxyl group so this value is pK_{a3}

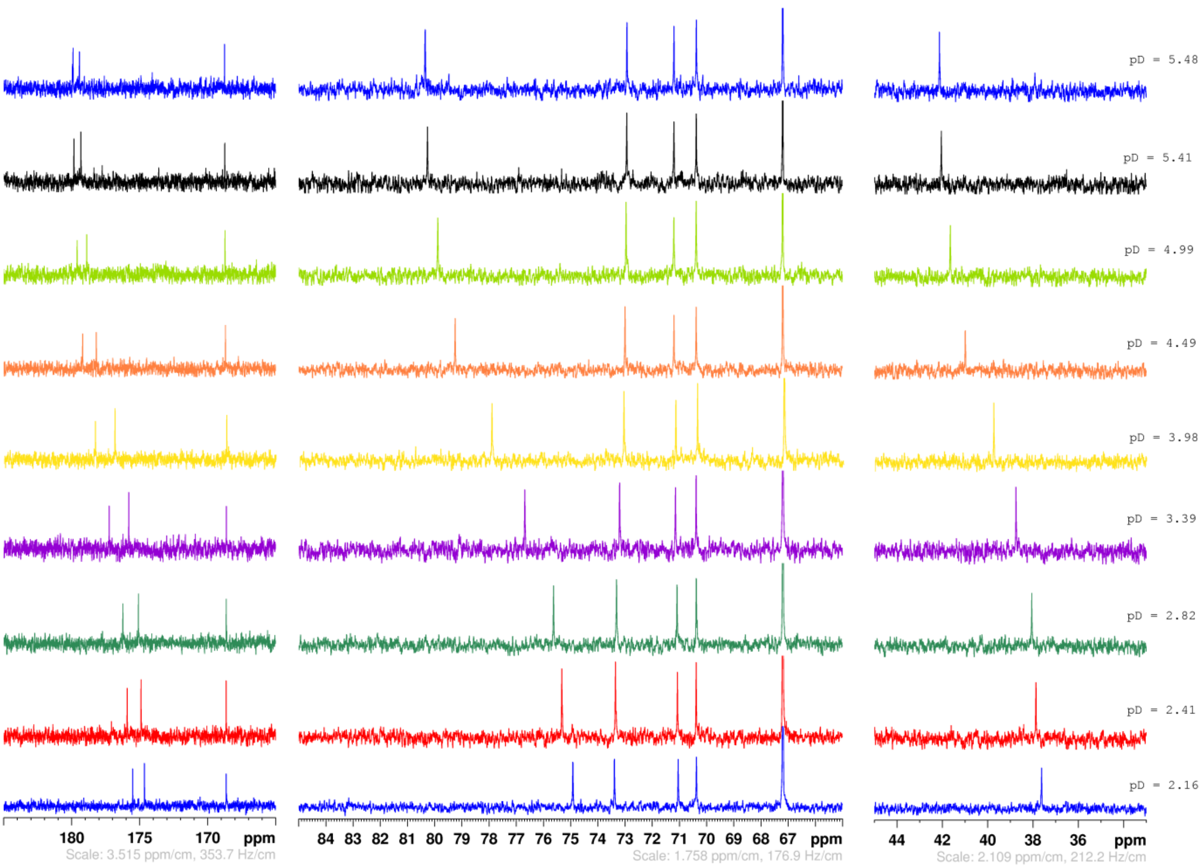

Figure S1. ^{13}C NMR analysis for malate pK_a determination of BSH. Overlay of selected regions of ^{13}C NMR spectra (100 MHz) at various pD.

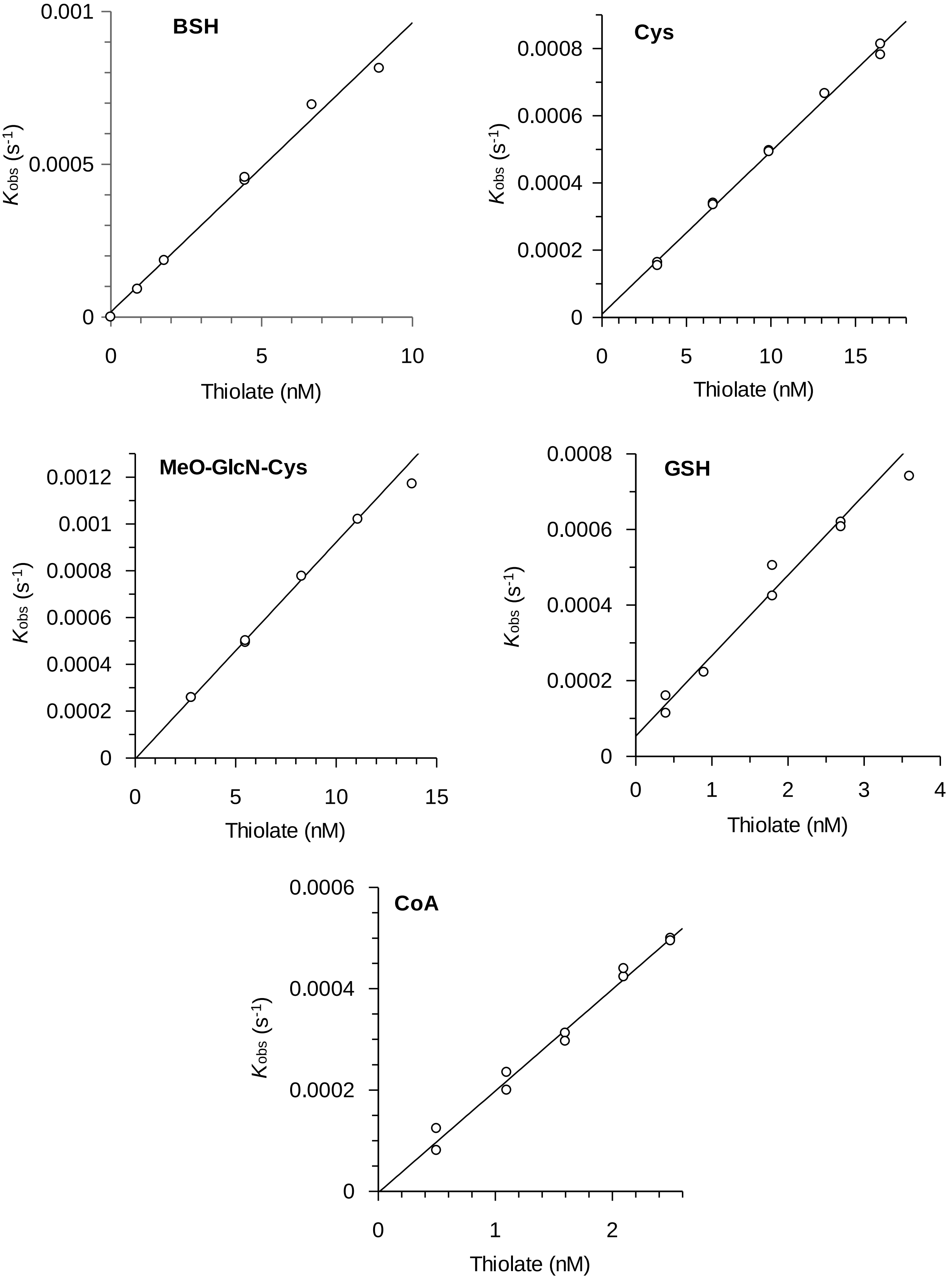

Figure S2. Plots of rate of TNB product formation (K_{obs}) *vs* thiolate anion concentration for different LMW thiols with DTNB.

Table S2. Thiol-disulfide equilibrium constants and redox potentials

Equilibrating species		Starting ratio [A]:[B]	K_{eq}	E^0 (mV)	Average E^0 (mV)
A	B				
BSSB	GSH	1:2	0.2866	-223.95	-221.0 ±3.1
BSSB	GSH	1:4	0.2750	-223.42	
BSH	GSSG	2:1	0.1864	-218.42	
BSH	GSSG	4:1	0.1836	-218.23	

Thiol and disulfide quantification in *B. subtilis*

Thiol analysis

Frozen cell pellets (corresponding to 5 mg rdw) were vortexed vigorously in 160 µL of 20 mM Hepes pH 8, 50% acetonitrile, 2 mM mBBr and then incubated at 60 °C for 15 min in the dark. After cooling on ice, methane sulfonic acid was added to a final concentration of 25 mM and the reactions were vortexed and centrifuged at 12,000 x *g* for 5 min. The pellets were dried overnight at 60 °C and weighed. The supernatant was filtered through a 0.2 µm membrane, and thiol samples were diluted five-fold with 10 mM methane sulfonic acid prior to analysis by HPLC.

Disulfide analysis

For disulfide analysis frozen cell pellets (corresponding to 30 mg rdw) were resuspended in 1 ml of 20 mM Hepes, pH 8, 50% acetonitrile, 5 mM NEM and then incubated at 60 °C for 15 min. After centrifugation at 12,000 x *g* for 5 min the supernatant was removed and 5 mM mercaptoethanol was added to the supernatant and incubated for 10 min at RT. The samples were concentrated to 100 µL in a speedivac and 2 mM DTT was added followed by 15 min incubation at RT. Subsequently 9 mM mBBr was added and incubated for 15 min at RT in the dark before adding methane sulfonic acid to a final concentration of 100 mM. The pellets were dried overnight at 60 °C and weighed. The supernatants were filtered through a 0.2 µm membrane, and diluted two-fold with 10 mM methane sulfonic acid prior to analysis by HPLC.

HPLC analysis for BSmB and CySmB (Method A)

BSmB and CySmB were separated by HPLC on a HiChrom ACE-AR C_{18} 4.6 x 250 mm, 5 µm, 100Å column, equilibrated at 37 °C with Solvent A (0.25% v/v acetic acid and 10% MeOH, adjusted to pH 4 with NaOH). Samples were eluted with a gradient of Solvent B (90% MeOH) at a 1.2 ml/min flow rate as follows: 0-5 min, 0% Solvent B; 5-15 min, 0-20% Solvent B; and 15-20 min, 20-100% Solvent B, followed by re-equilibration and reinjection. Detection was carried out with a Jasco fluorescence detector (FP-2020 Plus) with excitation at 385 nm and emission at 460 nm, and a gain of 1 x. BSmB and CySmB eluted at 11.8 min and 14.3 min, respectively.

HPLC analysis for CoAmB (Method B)

CoAmB was analysed by a different method using a Hichrom ACE C_8 column (4 x 150 mm, 5 µm, 100 Å) and a gradient of Solvent C (10 mM tetrabutylammonium phosphate (TBAP) in 90% water, 10% methanol, adjusted to pH 3.4 with acetic acid) and Solvent D (10 mM TBAP in 90% methanol, adjusted to pH 3.4 with acetic acid). The column was equilibrated with 30% Solvent D at 1 mL/min flow rate at 37 °C and the samples were eluted with the following gradient: 0-10 min, 30-50% Solvent D, 10-17 min, 50-100% Solvent D, followed by re-equilibration and reinjection. Detection was carried out with a Jasco fluorescence detector (FP-2020 Plus) with excitation at 385 nm and emission at 460 nm, and a gain of 1 x. CoAmB eluted at 15.4 min.
